# Post-Ischemic Renal Fibrosis Progression Is Halted by Delayed Contralateral Nephrectomy: The Involvement of Macrophage Activation

**DOI:** 10.3390/ijms21113825

**Published:** 2020-05-28

**Authors:** Pál Tod, Eva Nora Bukosza, Beáta Róka, Tamás Kaucsár, Attila Fintha, Tibor Krenács, Gábor Szénási, Péter Hamar

**Affiliations:** 1 Institute of Translational Medicine, Semmelweis University, 1094 Budapest, Hungary; todpal90@gmail.com (P.T.); nora.bukosza@gmail.com (E.N.B.); beata.roka@gmail.com (B.R.); kaucsar.tamas@med.semmelweis-univ.hu (T.K.); szenasi.gabor@med.semmelweis-univ.hu (G.S.); 2 Institute for Translational Medicine, Medical School, University of Pécs, 7624 Pécs, Hungary; 3 1st Department of Pathology and Experimental Cancer Research, Semmelweis University, 1085 Budapest, Hungary; fintha.attila@med.semmelweis-univ.hu (A.F.); krenacs.tibor@med.semmelweis-univ.hu (T.K.)

**Keywords:** acute kidney injury, chronic kidney disease, inflammation, MCP-1, TNF-α, mice

## Abstract

(1) Background: Successful treatment of acute kidney injury (AKI)-induced chronic kidney disease (CKD) is unresolved. We aimed to characterize the time-course of changes after contralateral nephrectomy (Nx) in a model of unilateral ischemic AKI-induced CKD with good translational utility. (2) Methods: Severe (30 min) left renal ischemia-reperfusion injury (IRI) or sham operation (S) was performed in male Naval Medical Research Institute (NMRI) mice followed by Nx or S one week later. Expression of proinflammatory, oxidative stress, injury and fibrotic markers was evaluated by RT-qPCR. (3) Results: Upon Nx, the injured kidney hardly functioned for three days, but it gradually regained function until day 14 to 21, as demonstrated by the plasma urea. Functional recovery led to a drastic reduction in inflammatory infiltration by macrophages and by decreases in macrophage chemoattractant protein-1 (MCP-1) and tumor necrosis factor-alpha (TNF-α) mRNA and most injury markers. However, without Nx, a marked upregulation of proinflammatory (TNF-α, IL-6, MCP-1 and complement-3 (C3)); oxidative stress (nuclear factor erythroid 2-related factor 2, NRF2) and fibrosis (collagen-1a1 (Col1a1) and fibronectin-1 (FN1)) genes perpetuated, and the injured kidney became completely fibrotic. Contralateral Nx delayed the development of renal failure up to 20 weeks. (4) Conclusion: Our results suggest that macrophage activation is involved in postischemic renal fibrosis, and it is drastically suppressed by contralateral nephrectomy ameliorating progression.

## 1. Introduction

Acute kidney injury (AKI) is an acute and serious reduction in kidney function diagnosed by an increase in serum creatinine and/or by decreased urine output (KDIGO) [1]. AKI is a frequent complication among hospitalized patients, but the true incidence of AKI is underestimated [2]. Patients with AKI have a higher risk to subsequently develop chronic kidney disease (CKD) and end-stage renal disease (ESRD) [3,4]. CKD is characterized by constantly declining glomerular filtration rate (GFR), the presence of various markers of kidney damage (e.g., proteinuria) and progressive fibrosis [5].

The leading causes of AKI are sepsis [6,7] and ischemia-reperfusion injury (IRI) [8,9]. Severe impairment of blood supply to the kidney triggers endothelial injury [10] and leads to the release of damage-associated molecular patterns (DAMPs), which increases the expression of monocyte chemoattractant protein-1 (MCP-1), also known as C-C motif chemokine 2 (CCL2). One of the main cytokines produced by macrophages is tumor necrosis factor-alpha (TNF-α). Thus, macrophage infiltration leads to increased TNF-α production [8,11,12,13]. Macrophage infiltration was reduced in mice deficient in C-C chemokine receptor type 2 (CCR2), the receptor of MCP-1. Additionally, these mice were largely protected from IRI-induced tubular necrosis [14]. Macrophages play a central role in the repair process following injury [15], but their sustained activation is a major contributor to the transition from repair to fibrosis. Especially in the setting of unilateral IRI, macrophages persisted beyond the time of repair, and MCP-1/CCR2 signaling played an important role in the cross-talk between injured tubular cells and infiltrating immune cells and myofibroblasts and promoted sustained inflammation and tubular injury with progressive interstitial fibrosis in the late stages of U-IRI [16]. The best-characterized profibrotic cytokine transforming growth factor-beta (TGF-β) activates myofibroblast formation and the production of fibroblast markers [17]. Myofibroblasts—characterized by alpha-smooth muscle actin (α-SMA) expression—are mainly responsible for the extracellular fibrotic matrix (fibronectin (FN) and collagen-1a1 (Col1a1)) deposition [18]. In summary, following severe AKI, the infiltrating macrophages play a central role in the transition of postischemic repair into progressive renal fibrosis characterized by glomerular sclerosis and tubulointerstitial fibrosis [3,19].

There are two distinct models of ischemic AKI in animals, the bilateral and the unilateral IR, induced by occluding the renal pedicles [11,20]. The contralateral kidney can remain intact [21] or can be removed either during the surgery [22,23] or later [24,25]. Surprisingly, delayed contralateral nephrectomy (Nx) induced partial functional recovery of the postischemic kidney, even following a severe (30-min) IRI, as described first by Finn WF [26] and, recently, by Skrypnyk et al. [24]. However, the mechanisms of functional recovery of the postischemic kidney are unknown. Furthermore, the roles of increased MCP-1 expression, consequent macrophage infiltration and enhanced TNF-α production after Nx have not yet been studied.

Our aim was to evaluate the magnitude and time-course of molecular mechanisms that contribute to the functional recovery of a postischemic and nonfunctioning kidney after Nx. For this reason, we investigated molecular events at various times up to three weeks after Nx. In a long-term experiment, we also followed the development of ESRD in the Nx group. ESRD of the postischemic kidney was delayed to about 140 days. Thus, delayed contralateral nephrectomy is an excellent model to study the mechanisms of renal fibrosis progression/reversal. Markers of renal fibrosis, oxidative stress and, especially, macrophage infiltration were drastically reduced by nephrectomy, suggesting a central role for macrophage activation in the development of ESRD in the postischemic kidney.

## 2. Results

### 2.1. Delayed Nephrectomy Almost Completely Restored the Function of the Postischemic Kidney

**Figure 1:**
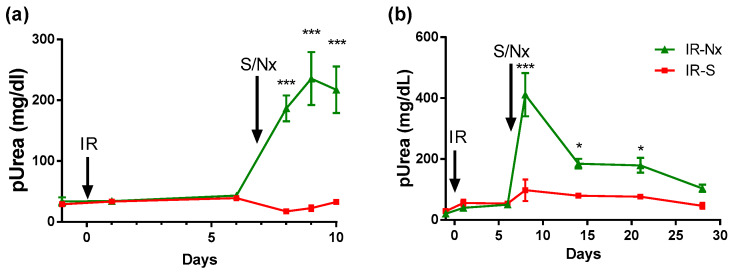
Effects of nephrectomy (Nx) on plasma urea concentrations in mice with left renal ischemia-reperfusion injury (IRI). (**a**) Blood samples were taken on days -1, 1, 6, 8, 9 and 10 in the 10-day study. (**b**) Blood samples were taken on days -1, 1, 6, 8, 14, 21 and 28 in the 28-day study. Data are expressed as mean ± SEM; two-way ANOVA with Tukey’s post hoc test; *: *p* < 0.05 and ***: *p* < 0.001; IR-S (sham) vs. IR-Nx.

Plasma urea concentration did not change after left IRI in mice with an intact contralateral kidney. Plasma urea sharply increased one day after Nx and remained elevated for at least three days (Figure 1a), suggesting that the postischemic kidney hardly functioned. However, plasma urea dropped sharply by day 14 and slowly decreased further by day 28. On day 28, plasma urea was similar in IR-Nx and the sham (IR-S) mice (Figure 1b). In the long-term study (see later), plasma urea was also normalized by Nx by day 28.

### 2.2. The Function of the Sole Postischemic Kidney Deteriorated Slowly

**Figure 2:**
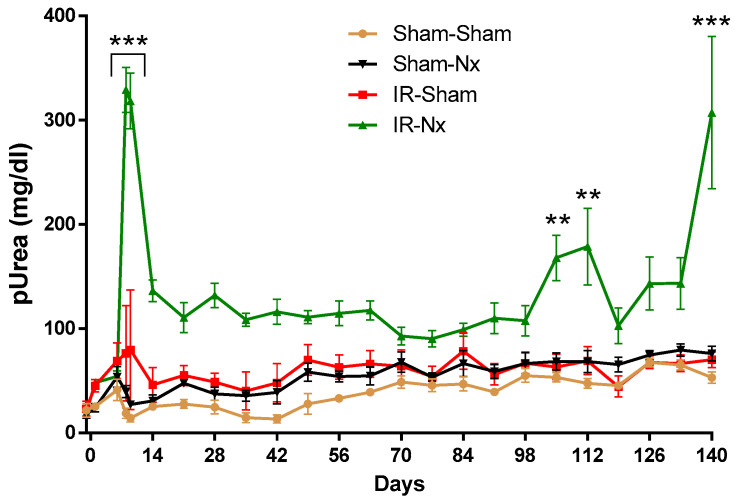
Plasma urea of mice in the long-term study. Samples were taken on days -1, 1, 6, 8, 9, 14 and once a week thereafter. Data are expressed as mean ± SEM; two-way ANOVA with Tukey’s post hoc test; **: *p* < 0.01 and ***: *p* < 0.001; IR-Nx (green) vs. all other groups.

A long-term study was conducted to follow the progression of CKD in the IR-Nx group in comparison to three control groups (S-S, S-Nx and IR-S). Two animals died two days after Nx in the IR-Nx group. Plasma urea did not change after Nx or sham operation in the S-S, IR-S and S-Nx groups, but thereafter, it rose slowly and gradually with increasing age of the mice (Figure 2). Plasma urea increased sharply one and two days after Nx in the IR-Nx group, but urea dropped by day 14 and slowly decreased further up to day 77 with some fluctuations. Plasma urea increased again after day 77 with a relatively long transient elevation from days 112 to 117. From day 133, plasma urea sharply increased again (Figure 2), and one mouse died on day 139, and three other mice were moribund (fatigue, eyes sunken and rough hair coat). Thus, the study was terminated on day 140, and the mice were sacrificed.

**Figure 3:**
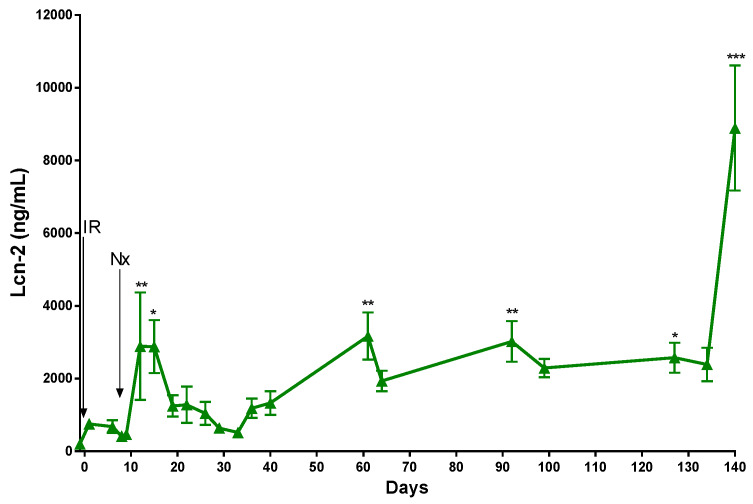
Plasma Lcn-2 of mice in the IR-Nx group in the long-term study. Data are expressed as mean ± SEM; one-way ANOVA with Tukey’s post hoc test; *: *p* < 0.05, **: *p* < 0.01 and ***: *p* < 0.001; day -1 vs. all other time points.

A significant increase in the concentration of the kidney injury marker lipocalin-2 (Lcn-2) was detected one and three days after Nx in the IR-Nx group. Lcn-2 gradually decreased thereafter and reached its minimum on day 33. Between days 33 and 127, the plasma Lcn-2 concentration steadily increased, with few transient elevations on days 66 and 100. A robust raise in the plasma Lcn-2 and plasma urea concentrations was detected simultaneously 20 weeks after IRI, when the mice were sacrificed (Figure 3).

### 2.3. Histology Demonstrated Partial Regeneration of the Postischemic Kidney after Nx

**Figure 4:**
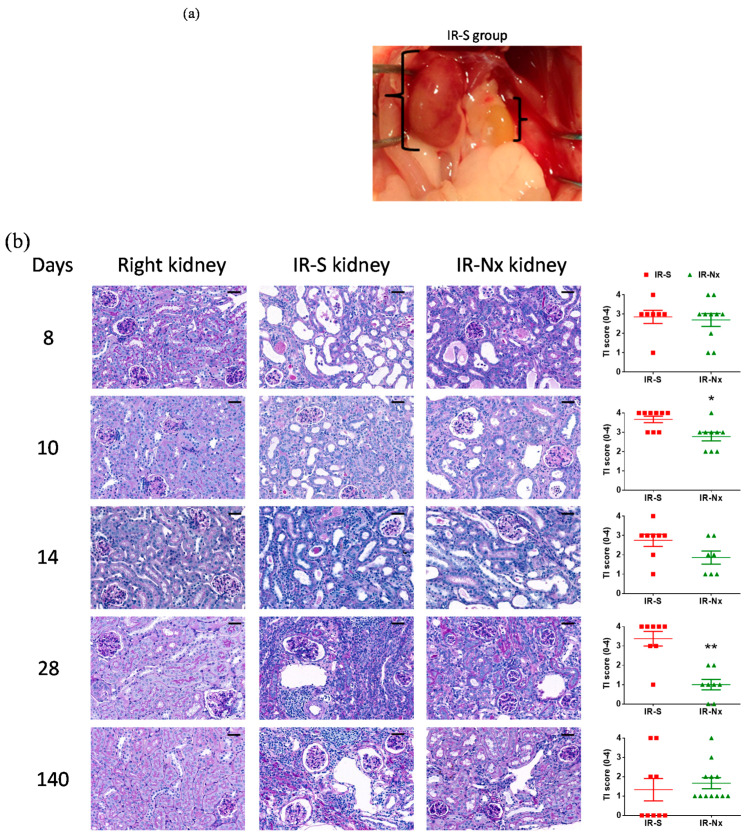

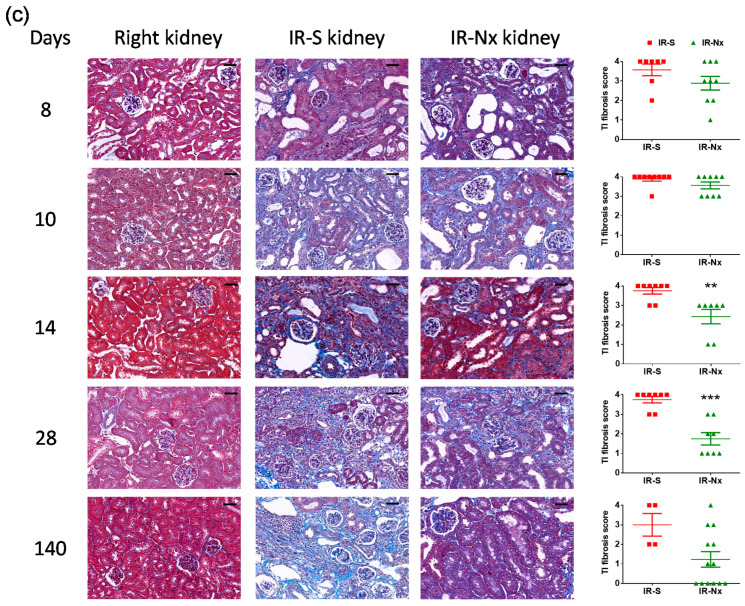
Kidney morphology. Representative pictures of nonischemic right (left column), postischemic left kidneys in the IR-S (middle histology column) and IR-Nx (right histology column) groups and the histology scores (right column). (**a**) Macroscopic picture of a nonischemic right and a pale, fibrotic/atrophic (postischemic) left kidney in the IR-S group on day 28 in situ. (**b**) PAS-stained sections (800×, scale bar: 50 μm) and tubular injury (TI) scores (0–4) (**c**) Masson’s trichrome-stained sections (800×, scale bar: 50 μm) and the tubulointerstitial (TI) fibrosis scores (0–4). Data are expressed as mean ± SEM; unpaired, nonparametrical Mann-Whitney *t*-test. *: *p* < 0.05, **: *p* < 0.01 and ***: *p* < 0.001 between the IR-S (red) vs. IR-Nx (green) groups.

Macroscopically, the postischemic kidneys were at least 50% smaller than the healthy contralateral kidneys on days 28 and 140 in the IR-S groups, and they appeared pale, suggesting hardly any blood perfusion (Figure 4a). On the other hand, the postischemic kidneys appeared normal in the IR-Nx groups.

With PAS staining, no histological alteration was seen in the nonischemic right kidneys (Figure 4b: right kidney (left) column). A marked tubular injury was seen in the postischemic kidneys demonstrated by loss of the brush border, flattening of the tubular epithelium, dilatation of the tubular lumen and PAS-positive material (hyaline) in the lumen of the distal tubules and collecting ducts. These alterations were present on days 8, 10 and 14 in all animals in most of the studied kidney surface area (>75%) in the IR-S groups. The tubular structures were lost to a large extent in the postischemic left kidneys on days 28 and 140 in the IR-S groups (Figure 4b: IR-S (middle histology column)).

A strong postischemic damage was present in the IR-Nx group on day 8; however, the kidney started to regenerate from day 10, as the tubular injury diminished by day 28 and was absent on day 140 in IR-Nx kidneys (Figure 4b: IR-Nx (right histology column)).

In IR-S kidneys, massive inflammatory infiltration of the tubulointerstitium was obvious from day 10 and, especially, from day 14. On days 28 and 140, massive tubulointerstitial inflammation was covering the tubulointerstitium (Figure 4b: IR-S (middle histology column)). However, tubulointerstitial inflammatory infiltration was absent in the IR-Nx group (Figure 4b: IR-Nx (right histology column)).

IR-S kidneys were atrophic already on day 28. The dilated pelvis and calyx were surrounded by thin, atrophic parenchyma with large vacuoles (Figure 4b: IR-S (middle histology column) and Supplementary Figure S1: IR-S column, day 28). Atrophy of the renal parenchyma was absent in the IR-Nx kidneys (Figure 4b: IR-Nx (right histology column)).

In Masson’s trichrome stained slides, the nonischemic right kidneys displayed red-stained healthy tubular epithelium without blue-stained tubulointerstitial fibrotic matrix deposition (Figure 4c: right kidney (left) column).

Tubulointerstitial damage with pathologic extracellular/interstitial matrix (ECM) deposition (blue-stained collagen in the tubulointerstitium) was obvious from day 7 in the postischemic left kidneys in the IR-S groups. Pathologic ECM was also present almost everywhere in the atrophic kidney in the IR-S group on day 140 (Figure 4c: IR-S (middle histology column)). Nx halted the extracellular matrix deposition as interstitial fibrosis was considrably reduced on days 14 and 28 in the IR-Nx group. However, interstitial fibrosis persisted to some extent even until day 140, as some blue-stained collagen was observed in the tubulointerstitium at all time points but only in a fraction of the fields (Figure 4c: IR-Nx (right histology column)).

Ischemia seldom caused glomerulosclerotic and vascular damage (data not shown).

**Figure 5:**
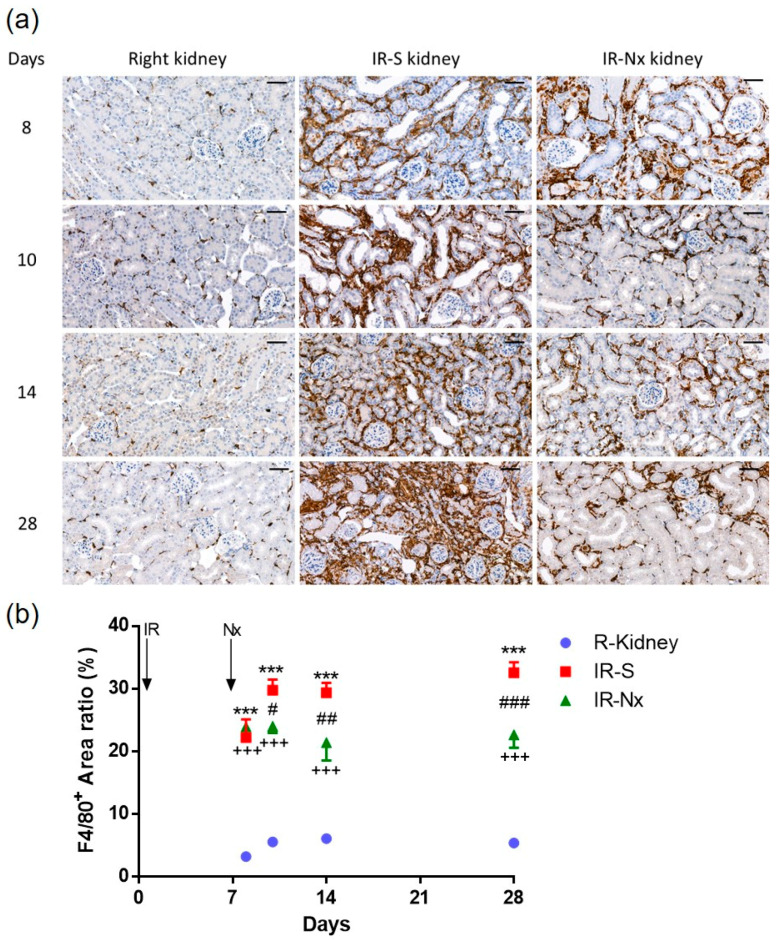
Macrophage-specific (F4/80) immunostaining of the kidney. Representative pictures of nonischemic right (left column) and postischemic left kidneys in the IR-S (middle column) and IR-Nx (right column) groups. (**a**) F4/80 staining (800x, scale bar: 50 μm). (**b**) Positive staining per whole kidney area ratio. Two-way ANOVA with Tukey’s post hoc test. ***: *p* < 0.001 IR-S (red) vs. nonischemic right kidneys (blue); +++: *p* < 0.001 IR-Nx (green) vs. nonischemic right kidneys (blue), #: *p* < 0.05, ##: *p* < 0.01 and ###: *p* < 0.001 IR-S (red) vs. IR-Nx (green).

A marked increase in macrophage infiltration was detected after IRI in all postischemic kidneys (IR-S: red and IR-Nx: green) at all studied time points, compared to the right kidneys (blue) (Figure 5a). However, macrophage content was reduced already on day 10 and remained lower up to day 28 in the IR-Nx groups (green) compared to the IR-S groups (Figure 5b) (red).

### 2.4. Nx Downregulated Inflammation-, Hypoxia- and Fibrosis-Related mRNAs in the Postischemic Kidney

**Figure 6:**
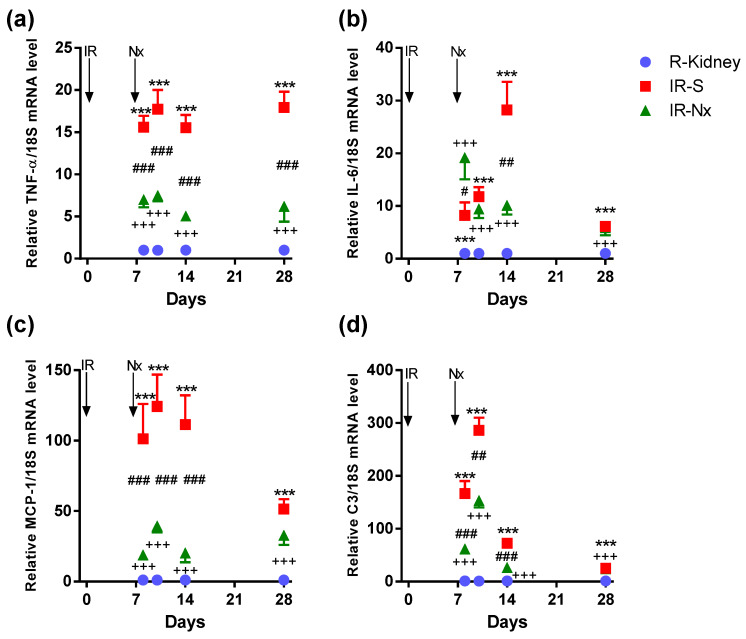
Fold changes in inflammation- and immune system-related mRNAs normalized to 18S. (**a**) TNF-α, (**b**) IL-6, (**c**) MCP-1 and (**d**) C3. Data are expressed as mean ± SEM; two-way ANOVA with Tukey’s post hoc test. ***: *p* < 0.001 IR-S (red) vs. nonischemic right kidneys (blue); +++: *p* < 0.001 IR-Nx (green) vs. nonischemic right kidneys (blue), #: *p* < 0.05, ##: *p* < 0.01 and ###: *p* < 0.001 IR-S (red) vs. IR-Nx (green).

**Figure 7:**
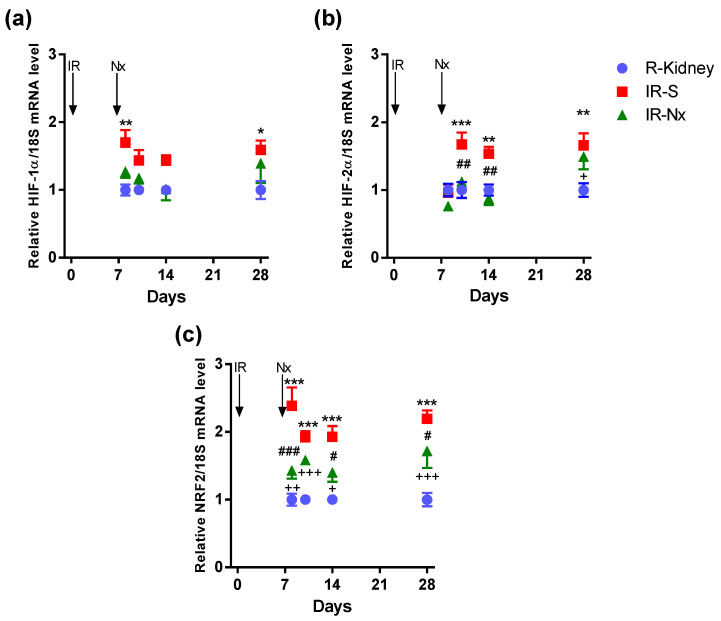
Fold changes in hypoxia- and oxidative stress-related mRNAs normalized to 18S. (**a**) HIF-1α, (**b**) HIF-2α and (**c**) nuclear factor erythroid 2-related factor 2 (NRF2). Data are expressed as mean ± SEM and two-way ANOVA with Tukey’s post hoc test. *: *p* < 0.05, **: *p* < 0.01 and ***: *p* < 0.001 IR-S (red) vs. nonischemic right kidneys (blue); +: *p* < 0.05, ++: *p* < 0.01, +++: *p* < 0.001 IR-Nx (green) vs. nonischemic right kidneys (blue), #: *p* < 0.05, ##: *p* < 0.01 and ###: *p* < 0.001 IR-S (red) vs. IR-Nx (green).

**Figure 8:**
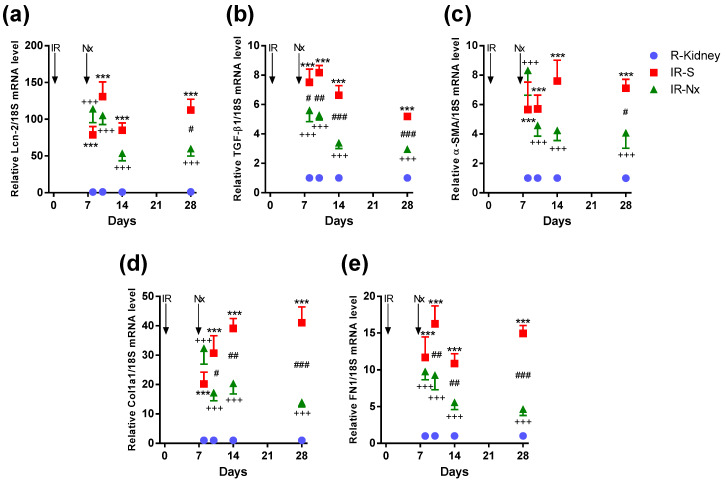
Fold changes in lipocalin-2 (Lcn-2) and fibrotic mRNAs normalized to 18S. (**a**) Lcn-2, (**b**) transforming growth factor-beta (TGF-β), (**c**) alpha-smooth muscle actin (α-SMA), (**d**) collagen-1a1 (Col1a1) and (**e**) fibronectin-1 (FN1). Data are expressed as mean ± SEM and two-way ANOVA with Tukey’s post hoc test. ***: *p* < 0.001 IR-S (red) vs. nonischemic right kidneys (blue); +++: *p* < 0.001 IR-Nx (green) vs. nonischemic right kidneys (blue), #: *p* < 0.05, ##: *p* < 0.01 and ###: *p* < 0.001 IR-S (red) vs. IR-Nx (green).

We analyzed mRNA expression of molecular markers in the kidneys on the first 28 days (Figure 6, Figure 7 and Figure 8). Kidney samples collected on day 140 were not investigated, as these postischemic kidneys were very fibrotic and atrophic. Our focus was to reveal the molecular background and driving forces that contributed to halting the progression of kidney damage and fibrosis. In the following sections, we describe first the mRNA expression measured in the postischemic IR-S kidneys (red) vs. the nonischemic right kidneys (blue) throughout the 28-day follow-up. Next, we describe the effect of Nx by comparing IR-Nx (green) vs. IR-S kidneys (red). The effect of Nx was never complete: as in the case of all investigated factors, there was a significant remaining effect of the ischemia despite the Nx when comparing IR-Nx (green) vs. sham (blue) kidneys.

Tumor necrosis factor-alpha (TNF-α, a main driver of inflammation and a main product of macrophages) mRNA was significantly and similarly elevated at all time points in postischemic IR-S kidneys (red) compared to the nonischemic right kidneys (blue). The maximum elevation was 18-fold on day 10. TNF-α mRNA stayed elevated at day 28 similarly to F4/80-stained macrophages but contrary to the other inflammatory cytokines (IL-6, C3 and MCP-1), which were much less elevated on day 28 than before. Nx significantly reduced TNF-α upregulation in all IR-Nx (green) vs. IR-S kidneys (red). Despite a significant reduction, TNF-α remained elevated at all time points in the IR-Nx (green) vs. the nonischemic right kidneys (blue) (Figure 6a).

Interleukin-6 (IL-6, a general proinflammatory cytokine) mRNA was significantly elevated on all days in the postischemic IR-S kidneys (red) compared to the nonischemic right kidneys (blue). The maximum elevation was 28-fold on day 14 with a sharp decline on day 28. Nx had the least influence on IL-6 upregulation from these cytokines and reduced only the peak of IL-6 upregulation in IR-Nx (green) vs. the IR-S kidneys (red) only on day 14. IL-6 was even higher in IR-Nx vs. IR-S on day 8. IL-6 also remained elevated at all time points in the IR-Nx (green) vs. the nonischemic right kidneys (blue) (Figure 6b).

Monocyte chemoattractant protein-1 (MCP-1) mRNA was significantly elevated in postischemic IR-S kidneys (red) at all time points compared to the nonischemic right kidneys (blue). The maximum elevation was 125-fold on day 10, with a sharp decline on day 28. Nx suppressed the whole MCP-1 peak at days 8–14 in IR-Nx (green) vs. IR-S kidneys (red) but had no more effect on day 28 when MCP-1 expression declined also in the IR-S kidneys (red). Despite a significant reduction, MCP-1 also stayed elevated at all time points in the IR-Nx (green) vs. the nonischemic right kidneys (blue). (Figure 6c).

Complement C3 (C3, an anaphylatoxin and mediator of inflammation and macrophage infiltration) mRNA was the most elevated among the mRNAs studied, with a 280-fold peak on day 10 in the postischemic IR-S kidneys (red) compared to the right kidneys (blue). Although C3 elevation peaked on days 8–10, its activation was the shortest, as its elevation was much less already on day 14, but was still elevated on day 28 in IR-S. Nx significantly reduced the whole C3 peak at days 8–14 in IR-Nx (green) vs. IR-S kidneys (red). Despite the significant reduction, C3 remained significantly elevated at all time points in the IR-Nx (green) vs. the nonischemic right kidneys (blue) (Figure 6d).

Hypoxia-inducible factor-1α (HIF-1α) mRNA was significantly elevated on days 8 and 28 only in postischemic IR-S kidneys (red) compared to the nonischemic right kidneys (blue). The maximum elevation was 1.7-fold on days 8 and 28. Nx prevented the small HIF-1α upregulation in IR-Nx (green) vs. IR-S kidneys (red), as HIF-1α was similar in all IR-Nx (green) and nonischemic right kidneys (blue) (Figure 7a).

HIF-2α mRNA was significantly elevated only from day 10 in postischemic IR-S kidneys (red) compared to the nonischemic right kidneys (blue). The maximum elevation was 1.7-fold on days 10 and 28. Similarly to HIF-1α, Nx prevented the small HIF-2α upregulation in IR-Nx (green) vs. IR-S kidneys (red), as HIF-2α was similar in all IR-Nx (green) and nonischemic right kidneys (blue) (Figure 7b).

Nuclear factor erythroid 2-related factor 2 (NRF2—a regulator of antioxidant protein expression) mRNA was significantly and similarly elevated at all time points in postischemic IR-S kidneys (red) compared to the nonischemic right kidneys (blue). The maximum elevation was 2.4-fold on day 8. NRF2 mRNA stayed elevated at day 28. Nx significantly reduced NRF2 upregulation at all time points except on day 10 in all IR-Nx (green) vs. IR-S kidneys (red). NRF2 remained elevated in the postischemic IR-Nx kidneys (green) compared to the nonischemic right kidneys (blue) only on days 8 and 28 (Figure 7c).

Lipocalin-2 (Lcn-2, also named as NGAL, a tubular injury marker) was significantly elevated at all time points in postischemic IR-S kidneys (red) compared to the nonischemic right kidneys (blue) with two peaks at days 10 (130-fold) and 28 (120-fold), suggesting an ongoing tubular injury after IRI. Nx reduced Lcn-2 upregulation in IR-Nx (green) vs. IR-S kidneys (red) only on day 28. Lcn-2 remained elevated in the IR-Nx (green) vs. the nonischemic right kidneys (blue) (Figure 8a).

Transforming growth factor-beta (TGF-β1, one of the main drivers of fibrosis) mRNA was significantly elevated at all time points in postischemic IR-S kidneys (red) compared to the nonischemic right kidneys (blue). The maximum elevation was eight-fold on day 10 and declined thereafter but stayed significantly elevated at day 28. Nx significantly reduced TGF-β1 upregulation in all IR-Nx (green) vs. IR-S kidneys (red). TGF-β1 remained elevated in the IR-Nx (green) vs. the nonischemic right kidneys (blue) (Figure 8b).

Alpha-smooth muscle actin (α-SMA, a fibroblast marker) mRNA was significantly and similarly elevated at all time points in postischemic IR-S kidneys (red) compared to the nonischemic right kidneys (blue). The maximum elevation was eight-fold on day 14 and was similar on day 28. Nx significantly reduced α-SMA upregulation on days 14 and 28 in IR-Nx (green) vs. IR-S kidneys (red). Despite a significant reduction, α-SMA was stayed elevated at all time points in the IR-Nx (green) vs. the nonischemic right kidneys (blue) (Figure 8c).

Collagen 1A1 (Col1a1, a main component of the fibrotic extracellular matrix) mRNA was elevated on day 8 and increased to day 28 in postischemic IR-S kidneys (red) compared to the nonischemic right kidneys (blue). The maximum elevation was 41-fold on day 28. Nx reversed Col1a1 upregulation to a progressively greater extent over time on days 10, 14 and 28 in IR-Nx (green) vs. IR-S kidneys (red). Despite the progressive reduction, Col1a1 remained elevated at all time points in the IR-Nx (green) vs. the nonischemic right kidneys (blue) (Figure 8d).

Fibronectin-1 (FN1, another main component of the fibrotic extracellular matrix) mRNA was significantly and similarly elevated at all time points in postischemic IR-S kidneys (red) compared to the nonischemic right kidneys (blue). The maximum elevation was 16-fold on day 10 and was similar on day 28. Nx significantly reduced FN1 upregulation in all IR-Nx (green) vs. IR-S kidneys (red), and—similar to Col1A1 production—the difference between the two groups progressively increased over time. Despite the progressive reduction, FN1 remained elevated at all time points in the IR-Nx (green) vs. the nonischemic right kidneys (blue) (Figure 8e).

## 3. Discussion

The main finding of the study is that the kidney becomes atrophic after unilateral ischemic injury in the presence of a healthy kidney. The postischemic kidney did not recover from the ischemic insult, as the plasma urea substantially increased for several days upon removal of the healthy kidney. Such a functional failure was related to increases in inflammatory, fibrotic and oxidative processes in the postischemic kidney. However, contralateral Nx induced a slow recovery of the postischemic kidney, leading to a drop in the plasma urea. In parallel, the expression of several pathogenic molecules, especially those of macrophage-driven inflammation drastically decreased. Similar studies have demonstrated previously that contralateral Nx at the time of renal ischemia [27] or two weeks later [28] can delay the progression of postischemic injury to end-stage renal fibrosis. However, these authors selected different time points for removal of the functional right kidney, and they did not investigate the time-course of progression and its amelioration by contralateral Nx or the long-term outcome with a late follow-up to demonstrate the development of fibrosis in the absence of a noninjured kidney.

Regarding the pathomechanisms behind the observed rapid fibrosis in IR-S mice and the halted progression following Nx in IR-Nx mice, we investigated inflammatory, hypoxia-driven and fibrotic processes.

We observed ongoing inflammatory processes in the postischemic kidney (IR-S group) at day 8 marked by TNF-α, MCP-1, IL-6 and C3 mRNA production, reaching their peak on day 10. The strongest upregulation was observed in the case of C3 (280-fold on day 10) and MCP-1 (125-fold on days 8–14). Although the C3 peak was the highest, it was also the shortest, as C3 upregulation diminished already by day 14. IL-6 and C3 elevations were much lower on day 28 than before. On the contrary, TNF-α remained as elevated as before even on day 28, and MCP-1 also remained significantly elevated, accompanied by strong inflammatory and F4/80+ infiltration throughout the observation period.

Thus, in postischemic kidneys after a contralateral sham operation, there was a strong complement (C3) upregulation. C3 as an anaphylatoxin is a potent proinflammatory mediator [29]. Although, C3 is produced mainly by hepatocytes [30,31], immune cells (including monocytes and tissue resident macrophages) can also secrete C3 [32]. Local C3 synthesis in the kidney has been linked to the progression of renal diseases [33,34]. C3 peak was the shortest, but—together with a sustained MCP-1 upregulation—macrophages were attracted to the inflamed kidney, as demonstrated by the F4/80 staining. Macrophages may be the main source of TNF-α in this setting, as C3, MCP-1, TNF-α and F4/80 infiltration reached their peak simultaneously on day 10, and both F4/80-positive cells and TNF-α and MCP-1 mRNA remained elevated on day 28, when the other inflammatory markers (C3 and IL-6) were fading away.

Prior to the functional recovery of the affected kidney, nephrectomy (Nx) decreased the expression of proinflammatory mRNAs (IR-Nx group). Based on the extreme elevations in C3, MCP-1 and TNF-α expression, we suspected a central role for macrophages. This hypothesis was supported by the extent of macrophage infiltration, as demonstrated by F4/80 staining. Following Nx, macrophage infiltration decreased in the kidney, strongly suggesting a pivotal role of macrophage-driven inflammation in ischemia-induced renal fibrosis. These observations are in-line with several previous results showing that, upon IRI, proinflammatory cytokines were significantly upregulated in the kidney [8,35,36]. Especially in the setting of unilateral IRI, persisting MCP-1 production was held responsible for sustained macrophage and myofibroblast infiltrations in the injured tubulointerstitium promoting fibrosis [16], supporting our conclusion that macrophages play a central role in this setting.

There is a long debate on hypoxia as a driving factor in the progression of CKD [37,38,39,40]. In our study, two isoforms of hypoxia-inducible factor were studied: HIF-1α and HIF-2α. At this late time interval (8–28 days), after the ischemic insult, the expression of these hypoxia-inducible factors did not seem to play a major role anymore, as their upregulation was only 1.7-fold. This observation suggests that hypoxia was mild when the kidneys regained their functional activity. It has been demonstrated, however, that HIFs exert their effect mainly by nuclear translocation [41], and their activity is not primarily regulated at the level of gene expression. Furthermore, Nx prevented HIF-1α and -2α upregulation, suggesting a pathogenic role of the ongoing mild HIF upregulation.

NRF2 elevation was somewhat higher (2.4-fold) than HIF and was similarly and constantly upregulated during the observation period, including day 28, suggesting a mild, ongoing oxidative stress in the kidney undergoing fibrosis (IR-S group). These observations are in-line with numerous previous studies, reporting protective effects of NRF2 in IRI [42]. Our findings also correlate with the results of Skrypnyk et al. [43], as a 28-day antioxidant treatment dose-dependently reduced fibrosis in the same animal model as used in our study.

In postischemic kidneys after a contralateral sham operation, profibrotic factors (TGF-β, α-SMA, Col1A1 and FN1) were already elevated by the beginning at day 8 and remained similarly elevated throughout the observation period, until day 28, suggesting an ongoing fibrogenesis. Nx significantly reduced the expression of all of these profibrotic factors but did not reduce them to control levels. Thus, ESRD developed in the postnephrectomy (IR-Nx) animals as well, but in 140 instead of 28 days.

Nx also reduced α-SMA mRNA from day 14, suggesting that myofibroblasts or their activity was reduced two weeks after Nx. Myofibroblasts are a main source of ECM deposition during fibrogenesis [44]. Accordingly, the progressively increasing effect of Nx on the production of extracellular matrix proteins (Col1A1 and FN1) suggests that Nx reversed the fibrotic matrix deposition. However, the already deposited matrix, as well as the sustained TGF-β and Col1A1 production by myofibroblasts, were enough to finally lead to ESRD. Similar ongoing fibrosis leading to ESRD was demonstrated by Chancharoenthana et al., who found high serum creatinine 20 weeks after 50-min IRI followed by delayed Nx in CD-1 male mice [45].

As macrophage infiltration-driven inflammation, as well as myofibroblast-driven matrix deposition and hypoxia, were significantly inhibited by Nx, the ongoing renal tubular damage was reversed, as demonstrated by Lcn-2, which began to decrease on day 14. Simultaneously, the excretory function of the postischemic kidney recovered, as demonstrated by continuously decreasing blood urea retention. Lcn-2 fluctuated in parallel with plasma urea during the study, and both markers increased sharply on the last week, indicating the development of renal failure.

In conclusion, severe unilateral ischemia-reperfusion injury with delayed contralateral nephrectomy offers a reproducible model to investigate the functional recovery of a nonfunctioning, fibrosing kidney. According to the results of our study, macrophage-driven inflammatory processes and subsequent reductions of the fibrotic matrix production and oxidative stress play important roles in the observed functional recovery and delayed progression to end-stage fibrosis.

## 4. Materials and Methods

### 4.1. Mice

Male Naval Medical Research Institute (NMRI) mice were purchased from Toxi-Coop (Budapest, Hungary). The animals were kept under standard conditions with free access to standard rodent chow (Akronom Kft., Budapest, Hungary) and tap water ad libitum. All protocols were approved by the Pest County Government Office and the Animal Ethics Committee of Semmelweis University (PE/EA/2202-5/2017). All experiments were performed in accordance with the Hungarian Acts XXVIII of 1998 and LXXVIII of 2018 on the protection and welfare of animals and EU Directive 2010/63/EU for animal experiments.

### 4.2. Experimental Design

Mice were anesthetized with i.p. injection of 80-mg/kg ketamine (Richter-Gedeon Nyrt., Budapest, Hungary) and 4-mg/kg xylazine (Streuli Pharma AG, Uznach, Switzerland). The body temperature of the animals was monitored during surgery with a rectal probe and kept at 37.0 ± 0.5 °C using a heating pad (Supertech Ltd., Budapest, Hungary).

**Figure 9:**
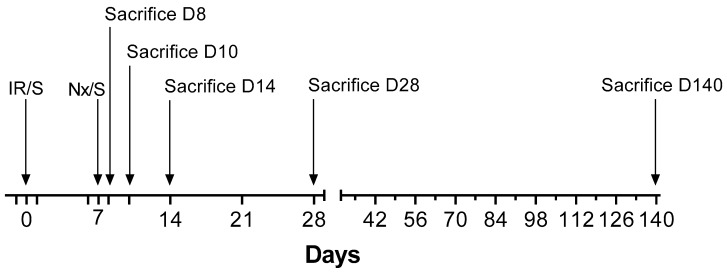
Overview of the interventions in each experiment of various durations. All major interventions are marked with arrow on the timeline. The time when blood and urine samples were taken after day 14 are marked as major and minor ticks, respectively.

We performed six separate experiments with a total of 96 animals based on the model published by Skrypnyk et al. [24] (Table 1). All mice underwent either 30-min ischemia/reperfusion (IR) or sham surgery (S) on the left kidney on day 0, a model regularly used in our laboratory [46,47]. Contralateral nephrectomy (Nx) or sham Nx was performed on day 7. Therefore, the following four types of groups were studied: (1) sham IR-Sham Nx (S-S), (2) Sham IR-Nx (S-Nx), (3) IR-Sham Nx (IR-S) and (4) IR-Nx. Separate experiments were terminated 7, 8, 9, 14, 28 or 140 days after the initial surgical interventions (Figure 9).

### 4.3. Blood and Urine Sampling and Sacrifice

Blood and urine samples were collected on days -1, 1, 6, 8, 9, 10, 14 and once weekly afterwards and on the day of termination (Figure 9). To prevent blood-clotting heparin (550 IU/mL, 10 mL/kg, i.p.) was administered 3 min before sacrifice. The animals were killed by cervical dislocation performed by a trained person. Blood was collected from the mice after cross-section of the vena cava superior. Mice were then perfused with 10-mL ice-cold physiological saline through the left ventricle.

Kidney samples were collected at nephrectomy (right kidneys) or at the time of termination (postischemic left kidneys). The upper-third part of the kidneys were placed in TRI Reagent^®^ (Molecular Research Center, Inc., Cincinnati, OH, USA) for RNA isolation. The middle section was cut at the hilus level, including all layers of the cortex and medulla (about 1-mm-thick cross-section) and fixed in 4% buffered formaldehyde (Molar Chemicals Ltd., Halásztelek, Hungary) for one day and then dehydrated and embedded in paraffin (FFPE) for histological analysis. The lower pole was snap-frozen in liquid nitrogen and kept at −80 °C for molecular studies.

### 4.4. Assay of Plasma Urea and Lipocalin-2 (Lcn-2) Concentrations

Plasma urea were measured from 2-μL undiluted plasma samples in 96-well plates using urea and glutamate-dehydrogenase enzymatic assay with colorimetric detection at 340 nm according to the manufacturer’s protocol (Diagnosticum Zrt., Budapest, Hungary). Plasma lipocalin-2 (Lcn-2) levels were measured at 450 nm with a mouse Lipocalin-2/NGAL DuoSet ELISA Development kit (R&D Systems, Minneapolis, MI, USA) after 1000–10,000-fold dilutions according to the manufacturer’s instructions. This kit detects natural and recombinant Lcn-2 in plasma and urine samples.

### 4.5. Total RNA Isolation and Real Time-Quantitative Polymerase Chain Reaction (RT-qPCR)

Total RNA was isolated using TRI Reagent^®^ according to the manufacturer’s protocol. Briefly, after phase separation using chloroform, the supernatants were aspirated and thoroughly mixed with isopropyl alcohol for RNA precipitation. The RNA pellets were washed twice with 70% ethyl alcohol and dissolved in RNase-free water (AccuGENE^TM^ Molecular Biology Water, Lonza, Basel, Switzerland). RNA concentrations were measured using Nanodrop 2000c Spectrophotometer (Thermo Fisher Scientific, Wilmington, DE, USA). RNA integrity was verified by electrophoretic separation on 1% agarose gel. Reverse transcription of 1-μg total RNA into cDNA was carried out using random hexamer primers and the High-Capacity cDNA Archive Kit (Applied Biosystem, Foster City, CA, USA) according to the manufacturer’s protocol. The mRNA levels of TNF-α, TGF-β, MCP-1, hypoxia inducible factor-1α and -2α (HIF-1α and HIF-2α), nuclear factor erythroid 2-related factor 2 (NRF2), α-SMA, FN1, Col1a1, interleukin-6 (IL-6), complement C3 (C3) and Lcn-2 were measured, and 18S mRNA was used for normalization (Table 2). All RT-qPCR was performed using SensiFast SYBR Green No-Rox kit (Bioline Reagents Ltd., London, UK) according to the manufacturer’s protocol.

### 4.6. Histology and F4/80 Immunohistochemistry

Renal tubular injury was evaluated on periodic acid-Schiff (PAS) and Masson’s trichrome-stained sections. Each tissue section was scored by a pathologist blinded to the experiment. Brush border loss, tubule dilatation and casts in the tubules were scored using the following scale: 0: no damage, 1: damage in 1–25% of the field, 2: 26–50%, 3: 51–75% and 4: >75%. Interstitial fibrosis was analyzed on Masson’s trichrome-stained sections, as described by Amman K et al. [48]. On this stain, collagen deposition in the extracellular matrix is stained blue, whereas cellular cytoplasm is stained red, and cell nuclei are dark-black. Interstitial fibrosis was scored using the following scale: 0: no, 1: 1–25%, 2: 26–50%, 3: 51–75% and 4: 76–100% of the field covered with interstitial fibrotic tissue.

For immunohistochemistry, the paraffin sections were mounted on Superfrost Ultra Plus Adhesion Slides (Thermo Fisher Scientific Inc, Waltham, MA, USA) and were deparaffinized and rehydrated in ethanol. The antigen retrieval was performed using Tris-EDTA solution (pH = 9). For nonspecific protein-blocking, 3% BSA (Millipore, Merck, Burlington, MA, USA) + TBST (Trisma base, sodium chloride, Tween 20 (Thermo Scientific, Waltham, MA, USA), pH = 4.4–7.6 were used. F4/80 immunohistochemistry was performed by incubating the slides with rabbit monoclonal anti-mouse antibody (F4/80 (D2S9R) XP(R) Rabbit mAb #70076S, 1:200; Cell Signaling, Leiden, The Netherlands) at room temperature for 2 h, followed by incubation with peroxidase-labeled anti-rabbit antibody (HistolS^®^-MR-T, Hisztopatológia Kft, Pécs, Hungary) at room temperature for 40 min. Color development was performed with diaminobenzidine (DAB) (Quanto, Thermo Scientific, Waltham, MA, USA). Slides were scanned with Pannoramic Scan (3DHistech Ltd., Budapest, Hungary) with a 20× objective × 10× lens (total magnification: 200×) with a 0.8-numeric aperture and then analyzed with Fiji ImageJ software [49]. F4/80-positive and total number of pixels were measured for each section, and their ratios were calculated.

### 4.7. Statistics

The results are expressed as mean ± SEM. The mRNA RT-qPCR data were analyzed after logarithmic transformation in case of significant inhomogeneity of variances indicated by Bartlett’s test. ROUT method [50] (significance level *p* = 0.01) was used to identify possible outliers, which were omitted from the analysis. One-way and two-way ANOVA were used for multiple comparisons followed by Dunnett or Tukey’s post hoc tests, respectively. For comparison of two groups, unpaired nonparametric Mann-Whitney *t*-test was performed using GraphPad Prism (version 6.01, GraphPad Software Inc, San Diego, CA, USA).

## Figures and Tables

**Table 1:** Number of animals involved in the studies performed. IR: ischemia/reperfusion, S: sham and N_x_: nephrectomy.

Study Duration	Groups			
	IR-S	IR-Nx	S-S	S-Nx
8 days	*n* = 7	*n* = 10	-	-
10 days	*n* = 9	*n* = 9	-	-
14 days	*n* = 8	*n* = 7	-	-
28 days	*n* = 8	*n* = 8	-	-
140 days	*n* = 4	*n* = 15	*n* = 5	*n* = 6

**Table 2:** The primers applied for qPCR.

Target Gene	Forward Primer	Reverse Primer
18S	CTCAACACGGGAAACCTCAC	CGCTCCACCAACTAAGAACG
α-SMA	TTCCTTCGTGACTACTGCCG	GCTGTTATAGGTGGTTTCGTGG
C3	ATCCAGACAGACCAGACCATCT	AGGATGACGACTGTCTTGCC
Col1a1	GACGCATGGCCAAGAAGACA	CATTGCACGTCATCGCACAC
FN1	CAGACCTACCCAGGCACAAC	CAGCGACCCGTAGAGGTTTT
HIF-1α	GGAGCCTTAACCTGTCTGCC	TGCTCCGTTCCATTCTGTTCA
HIF-2α	CCCTGCTGTCCTGCCTTATC	CATAGGCAGAGCGTCCAAGT
IL-6	CAAAGCCAGAGTCCTTCAGAGA	GGTCTTGGTCCTTAGCCACTC
MCP-1	TCACTGAAGCCAGCTCTCTCT	TCTTGTAGCTCTCCAGCCTACT
LCN-2	ACGGACTACAACCAGTTCGC	AATGCATTGGTCGGTGGGG
NRF2	CCTCACCTCTGCTGCAAGTA	GCTCATAGTCCTTCTGTCGCT
TNF-α	AAATGGCCTCCCTCTCATCA	AGATAGCAAATCGGCTGACG
TGF-β	CAACAATTCCTGGCGTTACCTTGG	GAAAGCCCTGTATTCCGTCTCCTT

## Supplementary Materials

Supplementary materials can be found at https://www.mdpi.com/1422-0067/21/11/3825/s1.
