# Supplementary material for: Post-Ischemic Renal Fibrosis Progression Is Halted by Delayed Contralateral Nephrectomy: The Involvement of Macrophage Activation
Source: Int J Mol Sci. 2020 May 28;21(11):3825. doi: 10.3390/ijms21113825 (PMC7312122; doi:10.3390/ijms21113825)
Supplement: Supplementary file 1 [file ijms-21-03825-s001.pdf]

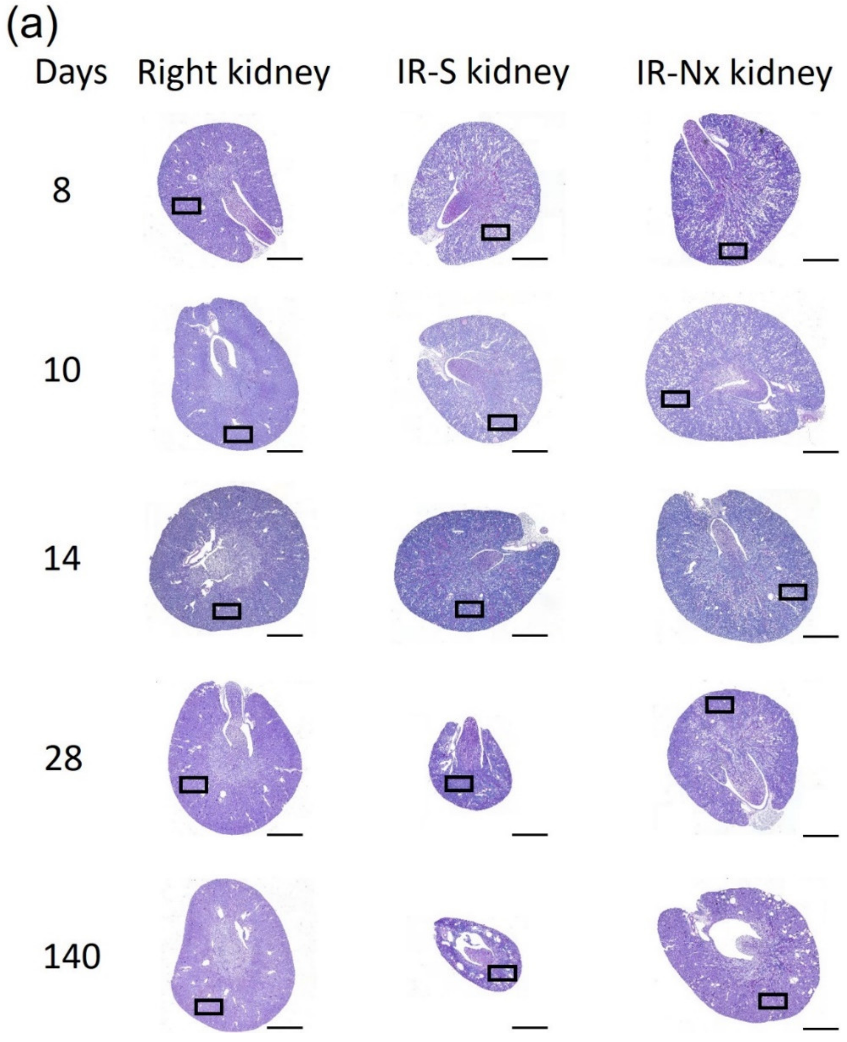
(a)
Days
Right kidney
IR-S kidney
IR-Nx kidney
8
10
14
28
140

(b)

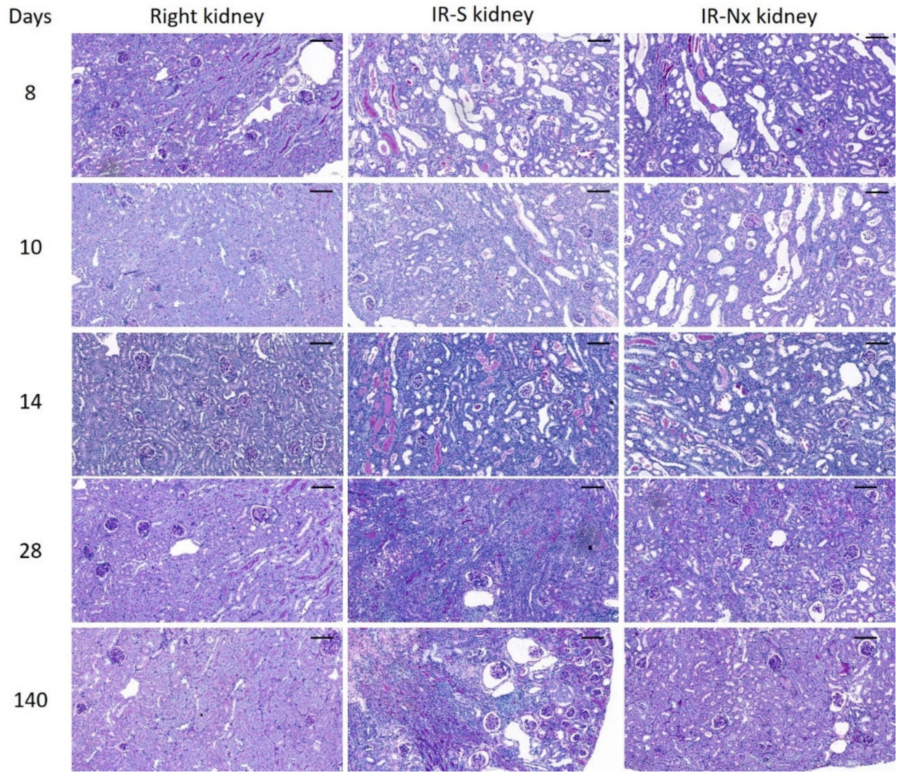

(c)

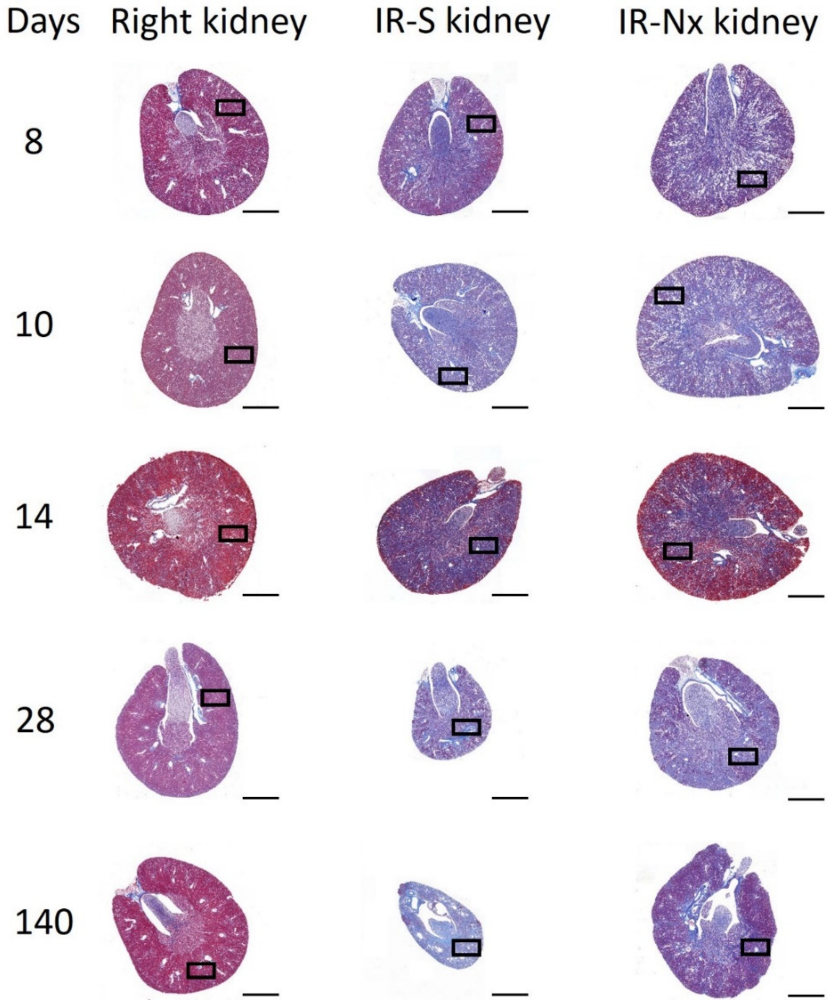

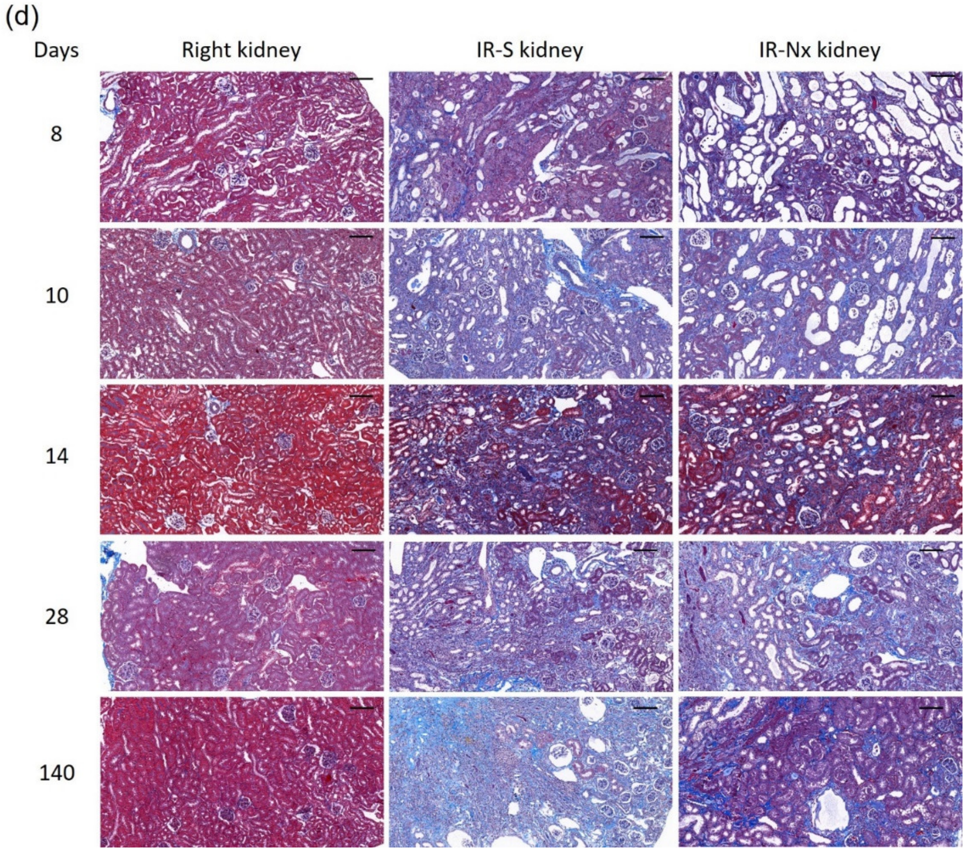
(d)
Days
Right kidney
IR-S kidney
IR-Nx kidney
8
10
14
28
140

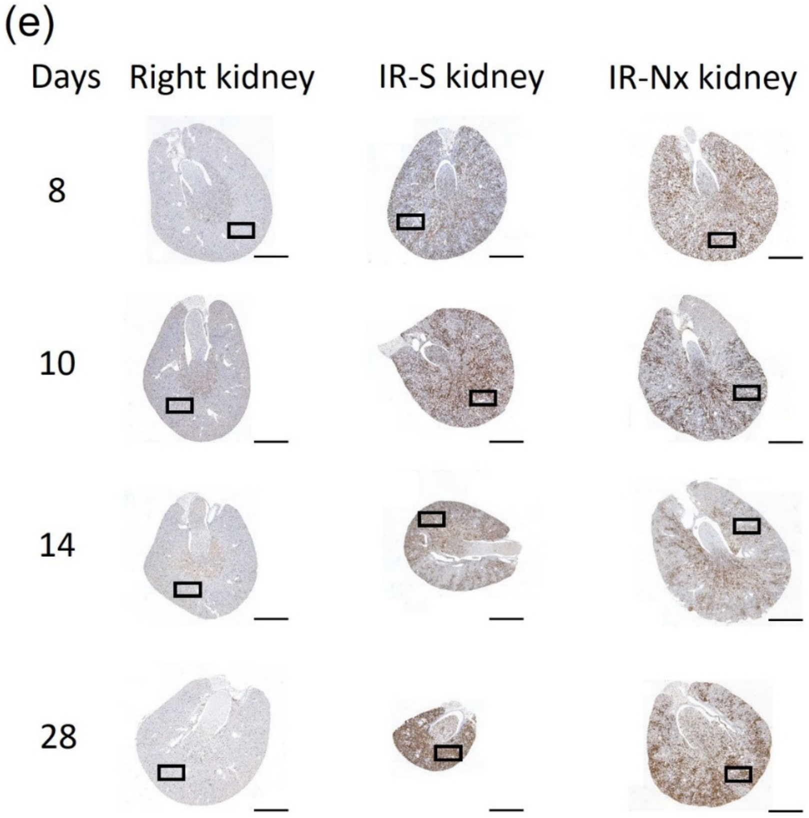

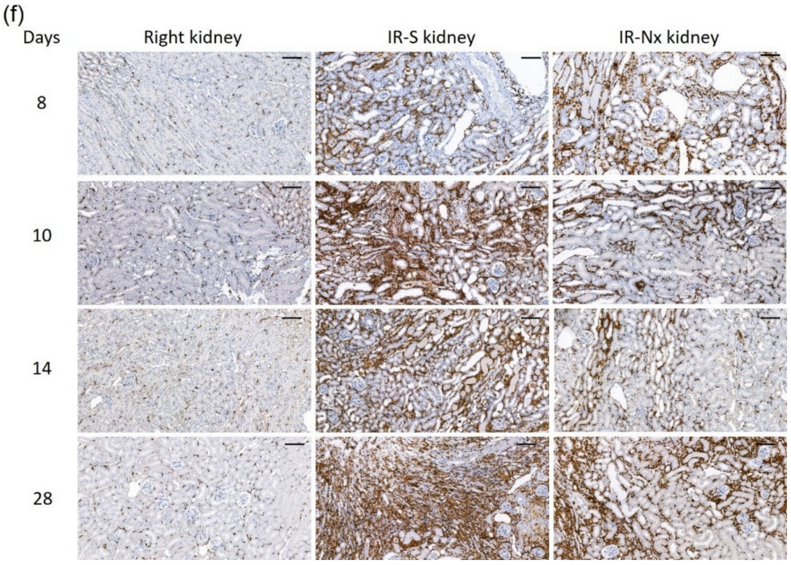

Supplementary Figure. S1. Representative pictures of kidney morphology and the macrophage-specific F4/80 staining of the kidney at various time points. (a), (b): PAS ((a): 40x, (b): 400x) (c), (d): Masson's trichrome ((c): 40x, (d): 400x). Scale bar: 1000 μm and 100 μm respectively. (e), (f): F4/80 ((e): 40x, (f): 400x). Scale bar: 1000 μm and 100 μm respectively. Non-ischemic right (left column), post-ischemic left kidneys in the IR-S (middle column)

and IR-Nx (right column) groups. Rectangle represents the kidney part from the 400x magnified pictures were taken.
